# Cumulative Family Risk and Internet Gaming Disorder Among Adolescents: A Serial Mediating Model of Personal Growth Initiative and Gratitude

**DOI:** 10.3389/fpubh.2022.819324

**Published:** 2022-04-11

**Authors:** Xiong Gan, Guo-Xing Xiang, Hao Li, Shao-Hua Wang, Xin Jin, Cong-Shu Zhu

**Affiliations:** ^1^Department of Psychology, College of Education and Sports Sciences, Yangtze University, Jingzhou, China; ^2^Yangtze University College of Technology and Engineering, Jingzhou, China

**Keywords:** cumulative family risk, internet gaming disorder (IGD), personal growth initiative, gratitude, serial mediating effect, adolescent

## Abstract

In the digital era, playing internet games enriches the entertainment forms for young generations. At the same time, it also raises some social issues, and internet gaming disorder (IGD) is one of them. Abundant studies demonstrate that IGD is harmful to individual physiological and psychological health. Therefore, it is necessary to figure out the reasons and mechanisms behind this phenomenon. Based on the ecological systems theory, the present study investigated the cumulative effect of family risks on adolescent IGD and the serial mediating effects of personal growth initiative (PGI) and gratitude in a chain mediation model. Using random cluster sampling, a sample of 600 Chinese adolescents was recruited to complete the questionnaire. Results of regression analysis suggested that cumulative family risks could positively predict IGD among adolescents. Moreover, PGI and gratitude mediated the relationship between cumulative family risk and IGD, separately, and sequentially. These findings may provide some guidance for the prevention and intervention to prevent or reduce IGD in adolescents.

## Introduction

In the digital era, playing internet games is one of the most popular forms of entertainment in modern society. By June 2021, the population of Chinese internet games had reached 509 million (1). Playing internet games in an appropriate way can effectively improve individuals' cognitive abilities (e.g., attention), emotional abilities (e.g., mood management), motivational abilities (e.g., resilience), and social abilities (e.g., prosocial behavior) (2). Moreover, a systematic review indicated that playing internet games can promote social wellbeing (3). Considering the popularity and benefits of internet games, they were developed as a formal sport and named “electronic sports,” which will be included in the Hangzhou (China) Asian Games 2022 as a formal medal event (4).

As the old saying goes, every coin has two sides. Playing internet games is not an exception. Uncontrollable, excessive, and compulsive use of internet games has become an important social problem, which is known as internet gaming disorder (IGD) (5). Abundant research has demonstrated that IGD has a significant influence on physiological and psychological health (6–8). Due to these serious consequences of IGD, the World Health Organization (WHO) considered it as a clinical disease in the latest version of the International Classification of Diseases (9). A meta-analysis across three decades indicated the alarming prevalence of IGD among adolescents (4.6%), especially among men (10). To prevent adolescents from suffering from IGD, researchers paid attention to the antecedents of this phenomenon. They revealed several predictors of IGD, such as self-esteem, attention problems, social vulnerability, and depressive symptoms (11–13). However, little research has examined the cumulative effect of various factors from the same subsystem, which may provide more effective guidance for practitioners to plan comprehensive prevention or intervention programs for IGD.

The ecological systems theory stresses that family is the most proximal microsystem for individuals and it has the most profound and persisting influence on individual development (14). Previous studies confirmed that numerous family risk factors were significantly associated with IGD among adolescents. For instance, Taechoyotin et al. (15) found that not living with parents significantly correlated with IGD among Thai adolescents. Similarly, Tullett-Prado et al. (16) reported that individuals with high IGD risk tended to live with divorced parents. Both of them indicate the potential association between family structure and IGD. Moreover, in a longitudinal study, Wichstrøm et al. (17) indicated that parents' education level could negatively predict adolescents' IGD 2 years later. As for the family financial situation, Faltýnková et al. (18) revealed that it had a negative association with internet addiction, of which IGD is a subtype. Thus, it is reasonable to believe that IGD might also have a close relationship with the family financial situation. Additionally, in a study of adolescents attending school, Bonnaire and Phan (19) indicated that adolescents with IGD experienced more family conflict and poorer family relationships, while adolescents without IGD had better family intimacy and cohesion. However, it is not difficult to realize that the relationship between IGD and these family factors is confirmed independently, which is not in line with reality to a certain degree. Adolescents may face these risks at the same time, and multiple risk exposures exceed the adverse developmental effects of singular exposures (20). Therefore, the present study will combine various family risk factors to examine their cumulative effect on adolescents' IGD. From a family aspect, this could provide a more effective framework for future prevention and intervention of IGD among adolescents.

### Mediating Effect of Personal Growth Initiative

Positive psychology focuses on the effects of positive factors on individual development. For a long time, personal growth initiatives (PGI) have received a lot of attention as an important topic in this domain. It refers to the individual tendency to consciously and actively improve oneself in the process of growth, which is a metacognitive construct and comprises two aspects, including cognitive and behavioral components (21). Previous studies demonstrated that, compared with those with a low level of PGI, individuals with a high level of PGI often behave more actively and are more likely to plan their lives and future, making them enjoy more subjective wellbeing, life satisfaction, and mental health (22–24). As a positive individual factor, PGI might also play an effective role in the relationship between cumulative family risks and IGD among adolescents. To date, no research has indicated the role of PGI in the aforesaid relationship, but some previous studies have provided indirect evidence supporting the potential mediating effect of PGI.

According to the ecological systems theory, family has a great influence on individual development (14), which includes the development of PGI as well. For instance, Whittaker and Robitschek (25) revealed that a multidimensional family function (e.g., family processes and family organization) could effectively predict PGI among college students. In a study of Japanese university students, Hirata and Kamakura (26) indicated that parenting style had significant effects on the level of PGI by improving or weakening self-esteem. Overall, PGI develops under the influence of the family subsystem and it will have profound effects on individual development. Prior studies found that PGI was negatively related to a series of problematic behaviors such as cyberbullying, alcohol use, deviant peer affiliation, and problem gambling (27, 28). As a problematic behavior, IGD may also correlate to PGI among adolescents. The risk-buffering model highlights that individual positive factors can effectively buffer or mitigate the deleterious impacts of environmental factors on individual development (29). Thus, considering the above indirect evidence and theories, the present study hypothesizes that PGI will mediate the relationship between cumulative family risks and IGD among adolescents.

### Mediating Effect of Gratitude

Gratitude, another important topic in positive psychology, can be conceptualized as an individual and generalized tendency to recognize the positive things obtained from the grace of others (30). Abundant evidence disclosed that gratitude was positively associated with positive outcomes such as life satisfaction, social support, prosocial behaviors, and subjective wellbeing (31–33). According to the risk-buffering model (29), as a positive individual factor, gratitude might also play a mediating role in the relationship between cumulative family risks and IGD among adolescents. The following previous studies have provided indirect evidence for the mediating role of gratitude.

Likewise, a gratitude tendency develops in the environment of a family system. So, it is ineluctable to be affected by family, which is confirmed repeatedly. In a study of Chinese elementary school students, Bai and Jin (34) revealed that family functioning was significantly correlated with gratitude. Moreover, family support was closely related to gratitude among middle school students (31). Similarly, parental support, especially maternal emotional support, sustains the level of gratitude among adolescents (35). Recently, Lam and Chen (36) demonstrated that college students with a healthy family interaction tended to develop a higher level of general gratitude. In a study of emerging adults, Lin (37) indicated that parenting effected their level of gratitude. Taking all these findings together, they confirm that the family subsystem plays an important role in the development of gratitude. Besides, prior studies demonstrated that gratitude had a significant relationship with IGD and other forms of addiction. For instance, in a study of Chinese left-behind children, Wei et al. (38) revealed that gratitude was negatively associated with IGD. Repeatedly, Wei et al. (39) indicated that gratitude could significantly predict less IGD through perceptions of school climate. Recently, Hou et al. (40) found that gratitude was negatively associated with internet addiction among college students. Given the theory and indirect evidence, the present study hypothesizes that gratitude will mediate the relationship between cumulative family risks and IGD among adolescents.

### PGI and Gratitude

Based on the risk-buffering model (29) and abundant indirect evidence, both PGI and gratitude might play mediating roles in the relationship between cumulative family risks and IGD among adolescents. Furthermore, previous studies suggested that there might be an association between these two mediators. In a study of Chinese gamblers, Loo et al. (27) demonstrated that PGI was correlated with gratitude, significantly and positively, and they both functioned as buffering factors for problem gambling. Moreover, Măirean et al. (41) and Voci et al. (42) repeatedly suggested that gratitude was positively related to personal growth. Besides, according to the self-determination theory (43), PGI can be considered as a kind of internal motivation, stimulated by which individuals will deliberately develop positive attributes such as gratitude. PGI underlines its metacognitive role in individual development (21), and gratitude is a more common tendency. So, gratitude might be included in the individual development influenced by PGI. Considering the above theories and empirical evidence, the family subsystem will significantly affect the development of PGI. With various levels of PGI, adolescents will obtain different motivations for developing gratitude, which sequentially will have a negative effect on IGD. Therefore, the present study hypothesizes that PGI and gratitude will serially mediate the relationship between cumulative family risks and IGD among adolescents.

### The Present Study

**Figure 1:**
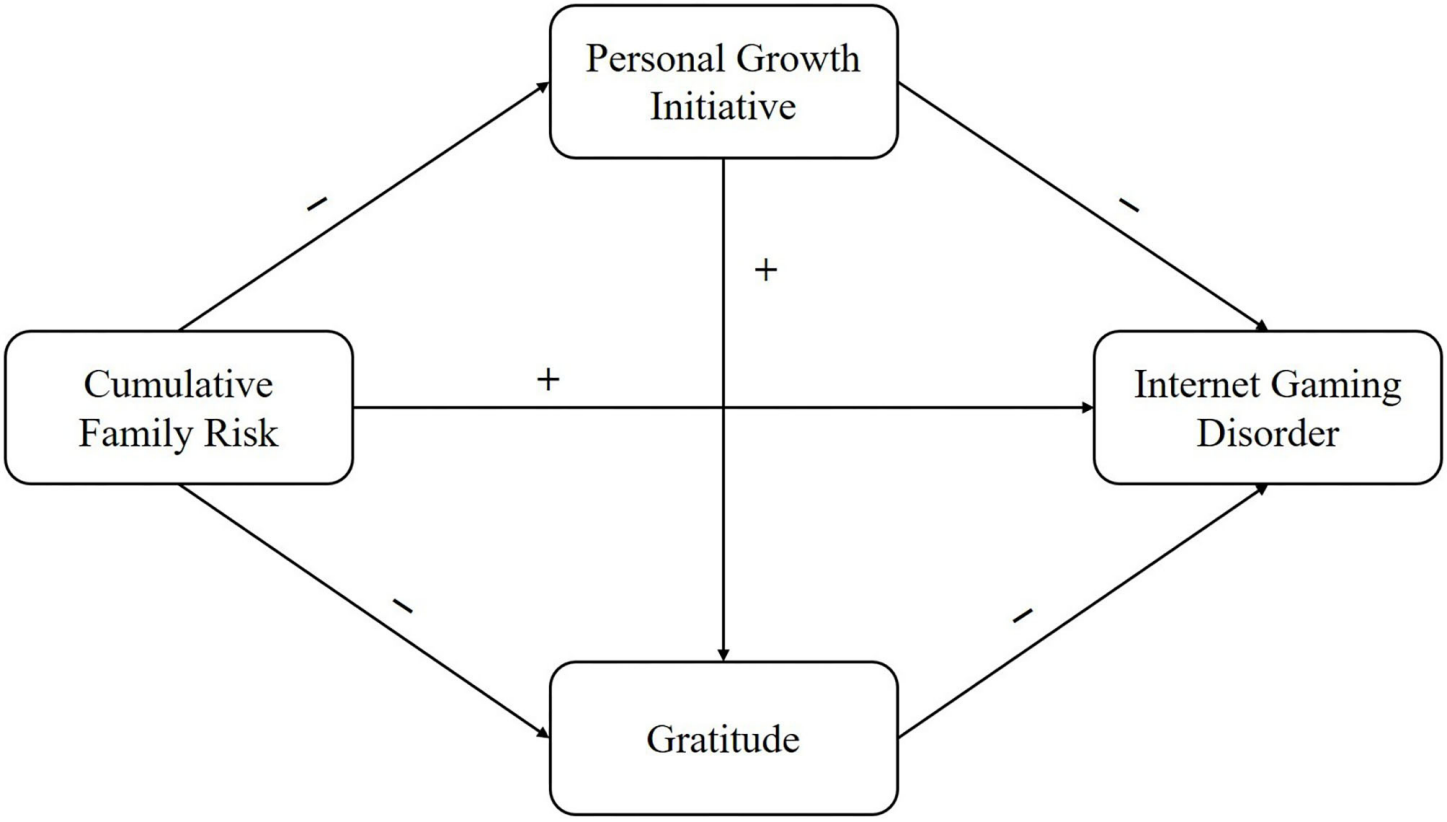
The hypothesized multiple mediation model.

Although IGD has been a research hotspot for a long time, to our knowledge, little research investigates the relationship between cumulative family risks and IGD from the perspective of positive psychology. With the assistance of empirical evidence and related theories, we realized the complicated associations between cumulative family risk, IGD, and other positive factors. Therefore, the present study attempts to assess the influence of cumulative family risks on IGD and the underlying mechanisms through a serial mediation model (Figure 1) with the following hypotheses: H1: PGI will mediate the relationship between cumulative family risk and IGD; H2: gratitude will mediate the relationship between cumulative family risk and IGD; H3: PGI and gratitude have a serial mediating effect on the relationship between cumulative family risk and IGD.

## Methods

### Participants and Procedure

Using random cluster sampling, 600 adolescents were recruited from four public middle schools in Hubei province and Hunan province in Central China. Among those, the mean age was 16.06 years (SD = 2.15) and 52.20% were boys. Before formal data collection, verbal informed consent was obtained from adolescents and their school administrators. Then, well-trained researchers administered the questionnaires to adolescents during school time. Meanwhile, honest responses were encouraged by informing them that their participation was voluntary and their answers were anonymous. Moreover, this survey was approved by the ethics committee of psychological research at the author's unit.

### Measures

#### Cumulative Family Risk

According to the six principles proposed by Li et al. (44) and the similar procedures used by other researchers (45, 46), the present study measured six family risk factors, including family structure, parents' education levels, family economic situation, family intimacy, family conflict, and parent–child relationship. These can be divided into three kinds of family risk factors, such as family structure, family resources, and family atmosphere. To a certain degree, they represent the most important family risk factors in the process of adolescents' development. In the present study, the cumulative family risk index was the sum of the recorded scores of these six factors. The following paragraphs describe the measurement details of these family risk factors.

##### Family Structure

Family structure was measured by one item “Do you live with both of your parents?” Responses were rated from “1 = yes” to “2 = no.” The response “yes” was recorded as zero risk and “no” indicated potential risk.

##### Parents' Education Levels

Parents' education levels were measured by the same two items, “what is your mother's or father's education level?” Responses were rated from “1 = primary school education” to “5 = master's degree or above.” Both parents with a high school education or above were recorded as zero risk. Even if only one parent obtained middle school education or less, it is considered a potential risk.

##### Family Economic Situation

The family economic situation was measured by the Family Economic Strain Scale (47). It consisted of four items rated on a five-point scale, from “1 = never” to “5 = always.” One example is that “my family cannot afford a nice house.” A higher score represented more family economic pressure. Scores higher than or equal to 75% were recorded as 1, indicating potential risks, and other scores represented zero risk. This scale demonstrated good reliability and validity among Chinese adolescents (47). In the present study, the Cronbach's α coefficient of this questionnaire was 0.84.

##### Family Intimacy

Family intimacy was measured by the family intimacy subscale from the Chinese version of the Family Adaptability and Cohesion Scales (FACES-II) (48). It contained 16 items rated on a five-point Likert scale from “1 = never” to “5 = always.” One example is that “in my family, every member can express his or her opinions freely.” A higher total score indicated a higher level of family intimacy. Scores lower than or equal to 25% were recorded as 1, indicating potential risks, and other scores represented zero risk. This scale demonstrated good reliability and validity among Chinese samples (48). In the present study, the Cronbach's α coefficient of this questionnaire was 0.85.

##### Family Conflict

Family conflict was measured by the conflict dimension from the Chinese version of the Family Environment Scale (FES) (48). It consisted of nine items rated from “1 = no” to “2 = yes.” One example is that “in my family, members often blame and criticize each other.” A higher total score indicated a higher level of family conflict. Scores higher than or equal to 75% were recorded as 1, indicating potential risks, and other scores represented zero risk. This scale demonstrated good reliability and validity among Chinese samples (48). In the present study, the Cronbach's α coefficient of this questionnaire was 0.67.

##### Parent–Child Relationship

The parent–child relationship was measured by the Parent–Child Relationship Scale (49). It contained nine items and was rated on a five-point Likert scale from “1 = never” to “5 = always.” One example is that “when you need to talk to your parents, they are glad to be a qualified listener.” A higher total score represented a better parent–child relationship. Scores lower than or equal to 25% were recorded as 1, indicating potential risks, and other scores represented zero risk. This scale demonstrated good reliability and validity among Chinese adolescents (49). In the present study, the Cronbach's α coefficient of this questionnaire was 0.89.

#### Internet Gaming Disorder

The 11-item Internet Gaming Disorder Questionnaire (IGDQ) (50) was used to measure adolescents' IGD. One example is that “do you need more time to play online games to feel satisfied?” All items were rated on a 3-point scale, ranging from “1 = never” to “3 = frequently.” The composite of IGDQ was the average score of all items, with higher scores indicating higher levels of IGD. This scale demonstrated good reliability and validity among Chinese adolescents (50). The Cronbach's α coefficient of this questionnaire was 0.89 in the present study.

#### Personal Growth Initiative

The PGI SCALE (21) was used to measure adolescents' PGI. It consisted of 9 items and was rated on a six-point Likert scale, ranging from “1 = totally disagree” to “6 = totally agree.” One example is that “if I want to change something in my life, I will start the transformation process.” A higher score indicated a higher level of PGI. This scale demonstrated good reliability and validity among Chinese adolescents (51). In the present study, the Cronbach's α coefficient of this questionnaire was 0.79.

#### Gratitude

The Gratitude Questionnaire-6 (52) was used to measure adolescents' levels of gratitude. It contained six items and was rated on a seven-point Likert scale, ranging from “1 = totally disagree” to “7 = totally agree.” One example is that “there are too many things in my life that I should be grateful for.” A higher score indicated a higher level of gratitude. This questionnaire demonstrated good reliability and validity among Chinese adolescents (53). In the present study, the Cronbach's α coefficient of this questionnaire was 0.80.

### Data Analysis

SPSS 26.0 and PROCESS macro were conducted to analyze the data. Initially, due to the way of data collection, the Harman single factor method was performed to assess the shared method biases. Second, descriptive statistics and bivariate correlations were conducted to analyze the association of main variables. Third, the linear regression test and model 6 in the PROCESS macro were used to examine the multiple mediation effects. Specifically, we conducted the linear regression test to assess whether cumulative family risk, PGI, and gratitude could predict IGD. Then, we constructed a serial mediation model to assess the mediating effects of PGI and gratitude on the relationship between cumulative family risk and IGD.

## Results

### Common Method Biases Analyses

Since the data were collected through self-reported questionnaires, the results might be affected by shared method bias. Therefore, the present study used questionnaires with reverse scoring and different rating scales. In addition, the Harman single-factor test was also adopted to assess the common method bias. The results showed that nine factors with a characteristic root >1, and the first factor explained 38.42% of the variation, which is much less than the critical value of 40%, suggesting that the shared method bias was not significant in the present study.

### Descriptive Statistics and Correlation Analyses

Table 1 shows the descriptive statistics of key variables for the current sample. As displayed in Table 2, all the main variables were significantly correlated with each other. Specifically, cumulative family risk was positively associated with IGD but negatively related to PGI and gratitude. Moreover, PGI was positively associated with gratitude, and both of them were negatively related to IGD.

**Table 1:** Descriptive statistics of key variables.

**Variables**	**Boys**		**Girls**		**Total**		
	**M**	**SD**	**M**	**SD**	**M**	**SD**	**Range**
CFR	2.84	1.83	3.11	1.85	2.97	1.84	0–6
1. Family structure	1.31	0.46	1.28	0.45	1.29	0.46	1–2
2.Parents' education levels	4.65	1.52	4.60	1.63	4.63	1.57	2–10
3. Family economic situation	7.46	3.61	7.41	3.67	7.44	3.64	4–20
4. Family intimacy	54.35	10.90	53.70	11.38	54.04	11.13	16–80
5. Family conflict	12.13	2.00	11.64	1.78	11.90	1.92	9–20
6. Parent-Child relationship	61.03	15.30	61.31	15.59	61.16	15.43	18–124
PGI	28.68	7.58	28.19	6.03	28.45	6.89	6–99
Gratitude	35.57	10.39	33.27	9.49	34.48	10.03	9–97
IGD	17.86	5.31	17.67	5.40	17.77	5.35	11–50

*CFR, Cumulative family risk; PGI, Personal growth initiative; IGD, Internet gaming disorder*.

**Table 2:** Skewness, kurtosis, and correlation coefficients of key variables.

**Variables**	**Skewness**	**Kurtosis**	**1**	**2**	**3**
1. CFR	0.43	−0.42			
2. PGI	2.78	31.09	−0.21^**^		
3. Gratitude	0.97	5.70	−0.20^**^	0.35^**^	
4. IGD	1.48	4.34	0.11^**^	−0.08^*^	−0.09^*^

*CFR, Cumulative family risk; PGI, Personal growth initiative; IGD, Internet gaming disorder*.

* *p < 0.05*,

** *p < 0.01*.

### Mediation Effect Analyses

**Figure 2:**
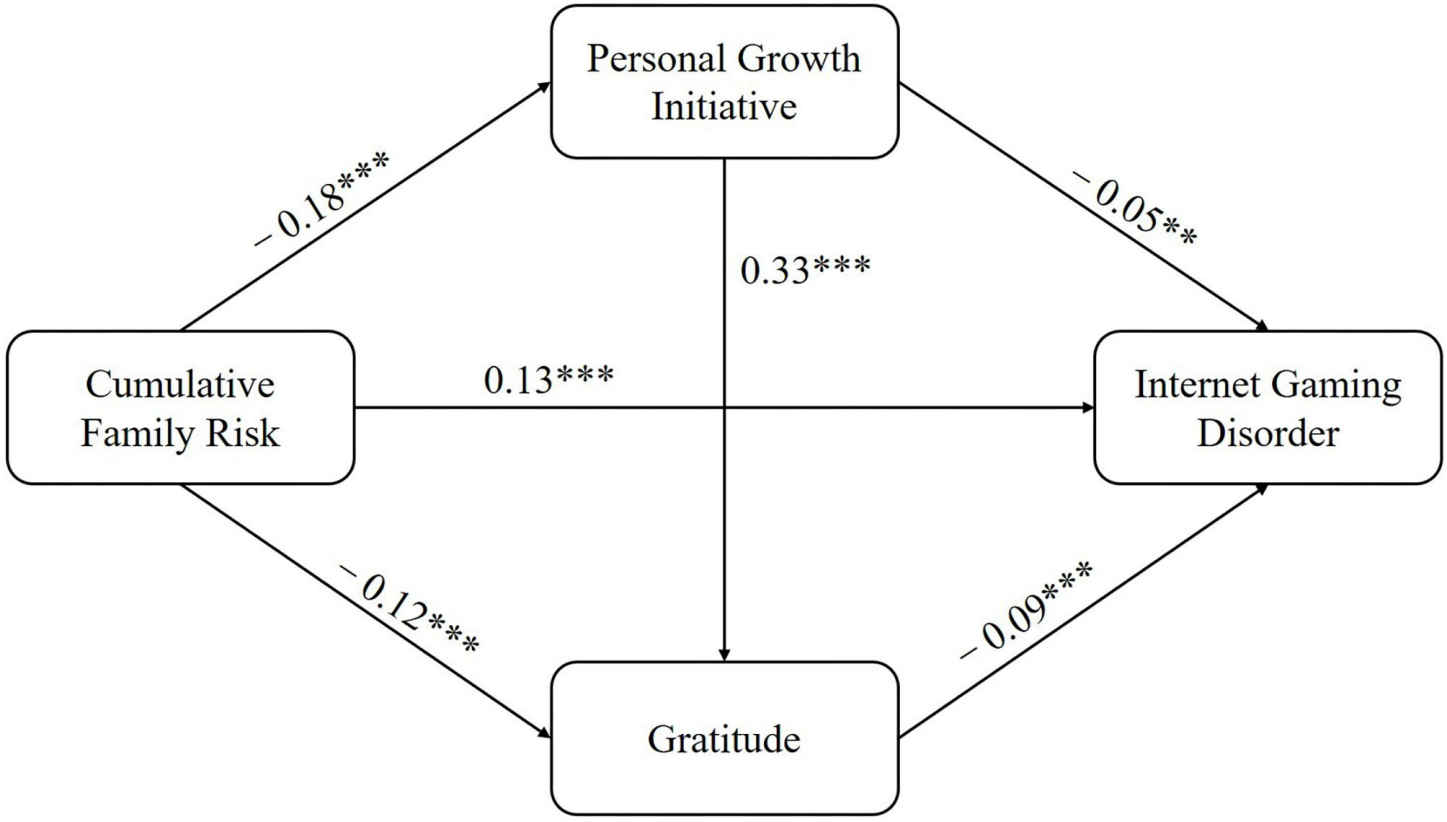
The multiple mediation model between cumulative family risk and IGD. **p < 0.01, ***p < 0.001.

After standardizing the main variables, a chain mediation model was constructed to examine the potential influences of PGI and gratitude on the relationship between cumulative family risk and IGD. With gender and age under control, the results of regression analyses (Figure 2) indicated that cumulative family risk could negatively predict both PGI (β = −0.18, *t* = −4.99, *p* < 0.001) and gratitude (β = −0.12, *t* = −3.42, *p* < 0.001), and PGI could also predict gratitude, significantly, and positively (β = 0.33, *t* = 8.84, *p* < 0.001). When taking all variables into consideration, cumulative family risk could predict a higher level of IGD (β = 0.13, *t* = 3.42, *p* < 0.001), while PGI (β = −0.05, *t* = −2.29, *p* < 0.01) and gratitude (β = −0.09, *t* = −2.37, *p* < 0.001) could predict a lower level of IGD.

Furthermore, bootstrapping analyses indicated that PGI and gratitude played mediating roles in the association between cumulative family risk and IGD. Table 3 displays the test results of indirect pathways. PGI mediated the link between cumulative family risk and IGD [β = 0.009, 95% CI = (0.02, 0.09)]. In more detail, cumulative family risk could predict a lower level of PGI, which further led to a higher level of IGD. Similarly, cumulative family risk could also result in more IGD by decreasing the level of gratitude [β = −0.011, 95% CI = (−0.10, −0.21)]. In terms of the chain mediation effect, results found that cumulative family risk could reduce the level of PGI, which could lead to less gratitude and then further increase the possibility of suffering from IGD [β = −0.001, 95% CI = (−0.01, −0.04)].

**Table 3:** Test results of indirect pathways.

**Indirect pathway**	**Effect size**	**Boot SE**	**95% CI**
1. CFR → PGI → IGD	0.009	0.010	[0.02, 0.09]
2. CFR → Gratitude → IGD	−0.011	0.006	[−0.10, −0.21]
3. CFR → PGI → Gratitude → IGD	−0.001	0.003	[−0.01, −0.04]

*CFR, Cumulative family risk; PGI, Personal growth initiative; IGD, Internet gaming disorder*.

## Discussion

Based on the ecological systems theory (14), the present study combined several family risks to investigate their joint effects on IGD among adolescents and also examined the underlying mediating mechanisms. Compared to the effect of a single factor, joint consideration of the cumulative risks seems more effective and practical to prevent adolescents from suffering IGD. The major findings of the present study were that PGI and gratitude had a serial mediating effect on the relationship between cumulative family risk and IGD. In the following paragraphs, the findings will be discussed in detail.

First of all, we revealed that cumulative family risk could predict a higher level of IGD in Chinese adolescents. That is, the more family risks adolescents experience, the more likely they will suffer from IGD. Previous studies indicated that some family risks, such as family type, parents' education, and family economic situation (15–18) were closely associated with IGD. The present finding repeatedly confirms these associations and further reveals their cumulative effects on IGD. Theoretically, this finding is consistent with the ecological systems theory that family greatly impacts individual development (14), reflecting not only the breadth but also the depth of those impacts. Practically, this finding from the family system suggests the complexity of the reasons for IGD, which indicates that measures should be taken to prevent and intervene in the phenomenon from a comprehensive perspective.

Consistent with the first hypothesis, PGI mediated the relationship between cumulative family risk and IGD among adolescents. In other words, experiencing too many family risks is harmful for adolescents' PGI, which in turn had fewer protective effects on their addiction to internet games. This finding supports the previous indirect evidence that PGI is significantly associated with family factors and problematical behaviors (25, 26, 28). In addition, this finding supports the opinion of the risk-buffering model that positive individual factors could buffer or mitigate the deleterious impacts of environmental factors on individual development (29), which suggests taking measures to help adolescents develop a higher level of PGI to avoid or decrease the effects of cumulative family risks on IGD.

Likewise, the second assumption was also verified. Gratitude worked as a mediator in the association between cumulative family risk and IGD. Cumulative family risks could increase the level of IGD by inhibiting gratitude tendency. Previous studies indicated that abundant family factors could promote the development of gratitude, such as family functioning, parental support, and family relationships (34–36). The current result reveals that lacking these factors will function as a risk factor for decreased gratitude. This supports those associations from another hand. Additionally, the current finding is consistent with other previous evidence that gratitude has a significant relationship with IGD (38, 39). Theoretically, the current finding also provides evidence for the risk-buffering model (29).

Another important finding is the serial mediating effect. Cumulative family risks could indirectly influence IGD through the sequential effects of PGI and gratitude. This result repeatedly confirms the association between PGI and gratitude, which was assessed in a study of the Chinese (27). The chain mediating effect highlights the metacognitive role of PGI, which could influence adolescents to develop a gratitude tendency. Moreover, this finding also supports the self-determination theory that PGI may be a kind of internal motivation to develop gratitude (43). In practical application, this finding is beneficial for the prevention and intervention of IGD. Specifically, compared with other factors, PGI is more influential. Thus, measures could be taken to help adolescents develop a higher level of PGI, which can not only buffer the harmful effects of family risks but also encourage adolescents to recognize or develop their gratitude tendency, thus serving a dual positive role.

In summary, the present study has the following strengths: First, risk factors from the family subsystem were taken together to assess their cumulative effect on adolescent IGD in the present study, which is more in line with the reality that multiple risks often come into existence together. In practice, comprehensive measures focused on the same subsystem could improve the effectiveness of intervention of IGD. Second, multiple mediating pathways were examined in the present study, which assists in making a comparison and choosing a more effective pathway for future prevention and intervention of IGD. Third, two positive factors, PGI and gratitude, were confirmed to function as buffers between family risks and IGD in the present study. To a certain degree, this further indicated the notion of positive youth development that adolescents have the attributes to meet the developmental challenges (54). Thus, future studies were encouraged to pay more attention to the positive attributes of adolescents.

Although the present study figures out the underlying chain mediators in the relationship between cumulative family risks and IGD among adolescents, it is not without limitations. In the present study, data were collected through self-reported questionnaires, which cannot avoid the social desirability problem. Moreover, the measures of family conflict and PGI did not demonstrate good reliability indices in the current sample. To obtain more objective and exact data, future research could use multiform measures, such as a combination of self-reported and other-reported questionnaires. In terms of the sample, the present study included Chinese adolescents. Future research could include samples from different cultures to obtain more generalized conclusions. As for the study design, individual development is a dynamic process, so future research could examine the current findings in a longitudinal study, which could come up with causal outcomes. Finally, the present study only investigated the role of the family system in adolescent IGD, whereas individual development is also influenced by other systems. Hence, future research could reconsider this issue in other systems such as schools and communities, as well as a combination of multiple systems.

## Data Availability Statement

The raw data supporting the conclusions of this article will be made available by the authors, without undue reservation.

## Ethics Statement

The studies involving human participants were reviewed and approved by Research Ethics Committee of College of Education and Sports Sciences, Yangtze University. Written informed consent to participate in this study was provided by the participants' legal guardian/next of kin.

## Author Contributions

XG designed the study. S-HW, XJ, and C-SZ collected and analyzed the data. G-XX drafted the manuscript. XG, HL, and G-XX revised the manuscript. All authors contributed to the article and approved the submitted version.

## Funding

This study was supported by Youth Project of Natural Science Foundation of Hubei Province in 2020 (2020CFB365) and Achievements of key projects of Education Science Plan of Hubei Province in 2019 (2019GA017).

## Conflict of Interest

The authors declare that the research was conducted in the absence of any commercial or financial relationships that could be construed as a potential conflict of interest.

## Publisher's Note

All claims expressed in this article are solely those of the authors and do not necessarily represent those of their affiliated organizations, or those of the publisher, the editors and the reviewers. Any product that may be evaluated in this article, or claim that may be made by its manufacturer, is not guaranteed or endorsed by the publisher.
